# Small Intestinal Obstruction with Intussusception due to Acute Myeloid Leukemia: A Case Report

**DOI:** 10.1155/2012/425358

**Published:** 2012-08-14

**Authors:** Sangeeta Kini, Anjali Amarapurkar, Meenaskshi Balasubramanian

**Affiliations:** Department of Pathology, BYL Nair Ch. Hospital, Mumbai 400008, India

## Abstract

Myeloid sarcoma is known to precede the development of acute myeloid leukemia (AML) and can be the only clinical manifestation. Gastrointestinal involvement by AML is rare with the commonest site being small intestine. Patients present with vague abdominal pain and/or obstruction. Prognosis is usually poor as most of them rapidly progress to AML. We report a case of 25-year-old man with complaints of abdominal pain and vomiting of one-year duration. OGD scopy revealed infiltration of lesser curvature of stomach. Subsequently patient came back within a week with signs and symptoms of acute intestinal obstruction for which an ileal resection was done. Although the histology of stomach biopsy and ileal segments showing similar features were thought to be non-Hodgkin's lymphoma, immunohistochemistry confirmed the diagnosis of myeloid sarcoma. Bone marrow investigations confirmed involvement by AML. Patient succumbed to the disease due to extensive involvement of AML. This case highlights the primary gastrointestinal manifestation of AML which can often prove to be a diagnostic difficulty clinically and histologically. Prompt diagnosis is essential to hasten the management.

## 1. Introduction

Myeloid sarcoma is a manifestation of acute myeloid leukemia (AML) with involvement of extramedullary sites, first described by Burns in 1811 [1]. Davey proposed the term extramedullary myeloid tumour [2]. The commonest sites of clinical manifestation of myeloid sarcoma are skin, bone/spine, and lymph nodes. Gastrointestinal involvement is not unusual, with the commonest site being ileum (10%-11%) followed by stomach and large intestine [3]. Clinical manifestation is often vague abdominal pain with or without obstruction, very rarely may present with perforation and bleeding. AML often develops subsequently within duration of 11months without chemotherapy [4]. We report a case of myeloid sarcoma primarily presenting with gastrointestinal manifestation which subsequently progressed to AML.

## 2. Case

A 25-year-old man presented with complaints of vague epigastric pain and vomiting of one-year duration. Radiological investigations revealed diffuse wall thickening along the lesser curvature with multiple enlarged lymph nodes in peripancreatic, paraaortic, and mesenteric group. Upper GI endoscopy revealed thickening of entire stomach especially lesser curvature. Rest of the routine blood investigation was within normal limits. Biopsy obtained from the stomach showed diffuse infiltration of mucosa and submucosa by neoplastic monotonous medium-sized cells with pale nuclei having occasional prominent nucleoli and brisk mitotic activity. Thus suspecting lymphoid cell morphology and a diagnosis of non-Hodgkin's lymphoma involving the stomach were given to be confirmed by immunohistochemistry.

Subsequently patient came back within a week with signs and symptoms of acute intestinal obstruction for which an emergency laparotomy was done and a segment of thickened ileum which had led to ileo-ileal intussusception was resected to relieve the obstruction. Grossly, a 4 cm long segment of ileum was received which showed mucosal thickening of 1.5 cm, and cut surface of the same showed diffuse white appearance.

**Figure 1:**
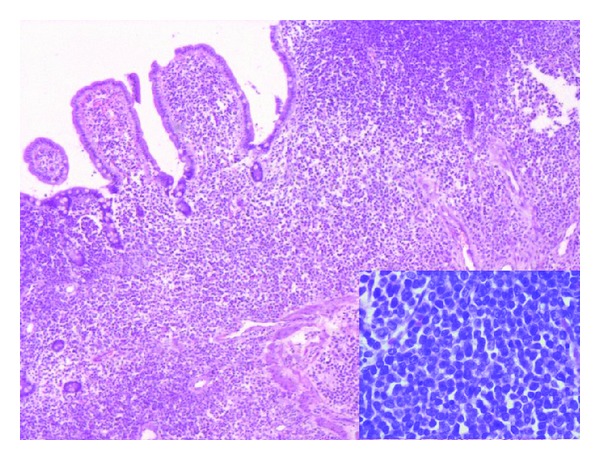
Section of intestine showing diffuse infiltration of mucosa and submucosa by neoplastic monotonous medium-sized cells. Individual cells have pale nuclei and occasional prominent nucleoli (Inset) ( H&E × 100X, Inset: H&E × 400X).

**Figure 2:**
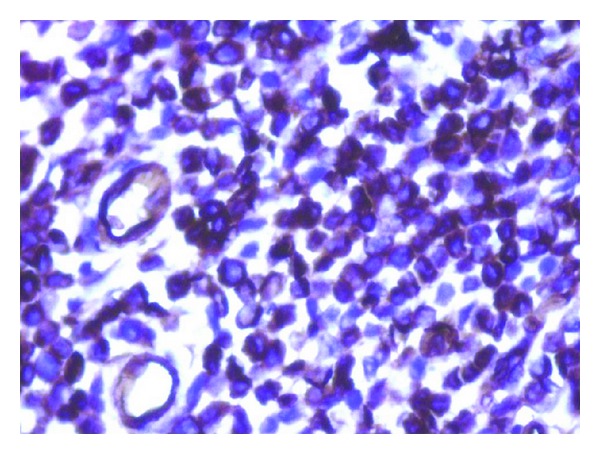
Section of intestine showing neoplastic cells strongly positive for CD34 (immunohistochemistry × 400X).

**Figure 3:**
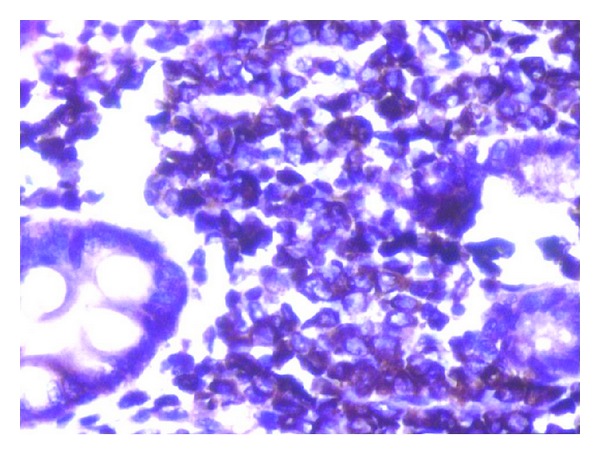
Section of intestine showing neoplastic cells strongly positive for MPO (immunohistochemistry × 400X).

Histology from the thickened ileum showed similar neoplastic monotonous cells of suspected lymphoid morphology as observed in the stomach (Figure 1). Considering clinico-pathological findings, a diagnosis of ileal involvement by non-Hodgkin's lymphoma was given. Immunohistochemistry performed on both stomach and ileum showed diffuse and strong positivity for CD34, MPO Ckit, MiB1 being 70% and negativity for CD3, CD20, and CD10 (Figures 2 and 3), thus making a diagnosis of extramedullary myeloid tumour or involvement by AML.

**Figure 4:**
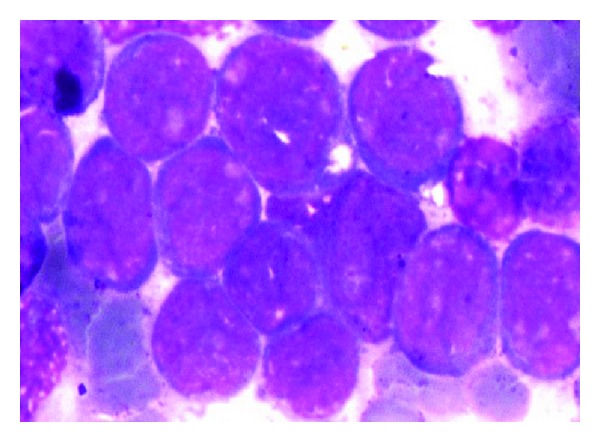
Bone marrow aspirate showing hypercellular marrow with 90% blasts morphology cells (Wrights × 1000X).

Subsequently, a bone marrow aspirate and biopsy done showed a hypercellular marrow containing almost 85%–90% cells of blast morphology, thus confirming marrow involvement by acute leukemia (Figure 4). Flow cytometry results showed CD34 98%, HLA-DR 98%, CD13 96%, CD33 99%, CD11 97% with negativity for CD10 and CD22 in 71% cells gated, thus confirming acute myeloid leukemia. Patient's condition deteriorated within 10 days of the postoperative period and succumbed to the disease due to extensive involvement by AML.

## 3. Discussion

Myeloid sarcoma (MS) can occur concomitantly or can precede AML in 2%–7% [3]. It can even present as a relapse form of treated AML. Myeloid sarcoma often remains underdiagnosed due to its variable clinical presentation. It can either present as an isolated tumour without hematological involvement or can present as apart of disseminated disease involving many organs. Among various subtypes of AML, M2 is commonly associated with the development of myeloid sarcoma. At least 90% of MS develop AML eventually in a period of 10-11 months [4, 5]. In our opinion, the present case although presented with myeloid sarcoma involving gastrointestinal involvement progressed rapidly to AML without any significant abnormalities in hematological parameters at the initial onset.

Common sites of MS involvement are skin, lymph nodes, spine, and so forth. Gastrointestinal MS although rare has been described both in adults and pediatric age group [6, 7]. Kohn et al. and Neiman et al. have described 7% to 11% of gastrointestinal MS; small intestine is the most common site followed by stomach and colon [3, 4]. Clinical diagnosis is often delayed due to wide spectrum of signs and symptoms such as vague acute or intermittent abdominal pain associated with nausea and vomiting due to partial or complete obstruction as noted in the present case. Very rarely presentation could be with signs/ symptoms of perforation or bleeding [3].

In the absence of abnormal hematological parameters, diagnosis of MS on histopathological specimens is challenging and 47%–56% of patients may be diagnosed with conditions other than MS, and the most common misdiagnosis being non-Hodgkin's lymphoma (large cell type) as observed in the present case, too [3, 8]. During differential diagnosis, the undifferentiated/blastic variant of MS needs to be differentiated from poorly differentiated carcinoma and melanoma in adults and neuroblastoma, ewing sarcoma and rhabdomyosarcoma in children. MS possesses similar immunohistochemical (IHC) profile as those of the blasts and precursor cells in AML, that is, MPO, CD34, CD 117, and chloroacetate esterase. Suspicion and applying this profile on all histopathological specimens including OGD scopy specimen is essential when lymphoma markers are negative. The treatment options of myeloid sarcoma of gastrointestinal involvement remain the same as that of AML that is, systemic chemotherapy with bone marrow transplantation in addition to surgical resection in appropriate clinical settings [3].

To conclude, myeloid sarcoma occurring at any site is the forerunner of AML and gastrointestinal MS involvement with most commonly to small intestine is a rare presentation due to its wide spectrum of clinical manifestations. As diagnosis of myeloid sarcoma in small intestine involvement is initially unrecognized, especially with the setting of normal-hematological parameters, suspicion in any mass with diffusely infiltrating tumour cells with right IHC profile is essential to hasten the appropriate management.
